# LoRa Mobile-To-Base-Station Channel Characterization in the Antarctic

**DOI:** 10.3390/s17081903

**Published:** 2017-08-18

**Authors:** Johnny Gaelens, Patrick Van Torre, Jo Verhaevert, Hendrik Rogier

**Affiliations:** Department of Information Technology, Ghent University/imec, Technologiepark-Zwijnaarde 15, 9052 Gent, Belgium; johnny.gaelens@UGent.be (J.G.); Jo.Verhaevert@UGent.be (J.V.); hendrik.rogier@UGent.be (H.R.)

**Keywords:** LoRa, antenna, propagation, measurement, Antarctic

## Abstract

Antarctic conditions demand that wireless sensor nodes are operational all year round and that they provide a large communication range of several tens of kilometers. LoRa technology operating in sub-GHz frequency bands implements these wireless links with minimal power consumption. The employed chirp spread spectrum modulation provides a large link budget, combined with the excellent radio-wave propagation characteristics in these bands. In this paper, an experimental wireless link from a mobile vehicle which transmits sensor data to a base station is measured and analyzed in terms of signal-to-noise ratio and packet loss. These measurements confirm the usefulness of LoRa technology for wireless sensor systems in polar regions. By deploying directional antennas at the base station, a range of up to 30 km is covered in case of Line-of-Sight radio propagation in both the 434 and 868 MHz bands. Varying terrain elevation is shown to be the dominating factor influencing the propagation, sometimes causing the Line-of-Sight path to be obstructed. Tropospheric radio propagation effects were not apparent in the measurements.

## 1. Introduction

Nowadays, low-power long-range networks are gaining interest in the context of the Internet of Things (IoT). Such networks are also very suitable for wireless communication with scientific measurement nodes in remote areas. In case of measurement setups in polar regions, systems need to operate at very low power. The increased scientific interest in live meteorological and seismic data in and around the Antarctic has given a boost to the research of very low power transmissions in extreme climate conditions.

In this paper, we study the channel conditions experienced by a communication system that monitors scientific experiments within a radius of 30 km around the Belgian Princess Elisabeth Antarctic Station [1]. The sensor nodes need to operate on battery power for at least a full year, as solar panels cannot be used during the long and dark winter. Snow accumulation on solar panels is also problematic. Solutions that need maintenance more than once a year are not feasible due to the difficulty and cost related to visiting scientific setups in the Antarctic.

For instruments near the station, omnidirectional antennas and commonly used modulation techniques perform well. This paper concentrates on scientific experiments deployed at a distance in the order of 20 to 30 km from the station. For these distances, modulation techniques with a larger link budget are preferred. For fixed scientific setups, the deployment of directional receive antennas delivers an obvious benefit. However, this setup is also advantageous for mobile applications, as the trajectory to the destination is likely to be along a straight line because of the absence of roads.

The Antarctic may seem endless and flat, but for the region (Queen Maud Land) where this research is being performed, measurements show that for expeditions north of the Princess Elisabeth base at distances of less than 10 km, the slope of the ice already causes an absence of Line-of-Sight (LoS) conditions. For expeditions to the south, mountain tops are preventing this straight-line trajectory, making wireless data transmission very difficult but not impossible, as experimentally observed. Another important aspect is the total absence of other transmitters in the area, causing the frequency band to be exceptionally free of interference. This is also reflected in the absence of spectrum regulations for the Antarctic region.

In this paper, channel measurements are presented for wireless mobile-to-base-station links in the Antarctic. The results provide valuable insights to scientists planning the deployment of fixed point-to-point wireless links from their sensors to a base station. In such a scenario, the communication channel is unidirectional, with the aim of wirelessly transmitting sensor data from the sensor to the base station at minimal power. Sub-GHz frequencies may still provide a long communication range in such operating conditions. The wireless communication system relies on LoRa technology [2], whose chirp spread spectrum (CSS) modulation provides a large link budget. CSS modulation yields spreading gain, accurate channel estimation, and suppression of delayed multipath components. Transmissions are performed at 434 and 868 MHz, such that propagation characteristics at these frequencies can be compared. The received signal levels are recorded as a function of distance to the base station. Terrain elevation is also taken into account.

Although many channel measurements and models [3] are documented for rural and urban environments in the 700–900 MHz frequency range, only a scarce number of publications about LoRa channel characteristics exist at the moment, as this is a relatively new technology. Measurements for fixed setups over a 700-m range in a suburban environment are described in [4]. The authors of [5] have performed outdoor measurements to determine the link budget for a range up to 8 km. Therefore, they have employed LoRa hardware by the Semtech corporation, similar to the hardware applied in this manuscript. Another field test [6] compares LoRa specifications to other standards operating in the sub-GHz frequency bands, such as Sigfox [7] and DASH7 [8]. Ref. [9] describes outdoor and indoor lab measurements to determine Doppler robustness, scalability, and coverage. Interestingly, the latter paper obtains reasonable performance up to 30 km range over water, which is comparable to the range observed in our measurements over snow and ice. Ref. [10] evaluates interference when performing LoRa transmissions in the 868 MHz band. Crowded environments suffer from high interference probabilities, resulting in performance degradation. LoRa scalability tests—also based on interference measurements—are analyzed more thoroughly in [11]. The paper is further organized as follows. The equipment used for the measurements is documented in Section 2, followed by the measurement results in Section 3. The discussion and conclusions follow in Section 4 and Section 5.

## 2. Materials and Methods

The measurement campaign is based on a setup employing a fixed LoRa transceiver with directional antennas at the base station and a mobile transceiver equipped with two omnidirectional antennas deployed on a moving 4×4 vehicle. The snowmobile is a Toyota Hilux equipped with tracks, resulting in an antenna height of 210 cm above ground level.

**Figure 1:**
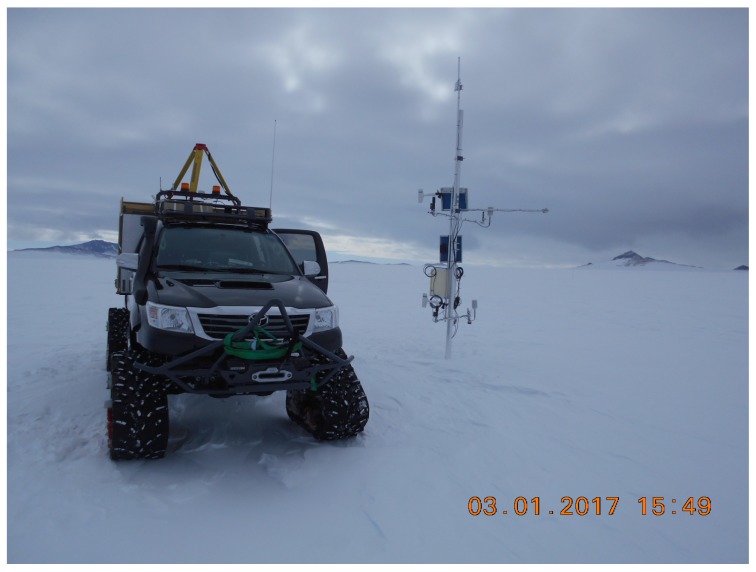
Mobile, carrying out the channel characterization experiments at one of the fixed wireless sensor nodes, monitoring meteorological conditions in the Antarctic.

We study a unidirectional channel where the mobile transmits sensor data to the base station at minimal transmit power. The knowledge obtained about such a wireless channel will help scientists to optimally deploy fixed wireless sensor nodes that monitor the Antarctic environment. Current sensor systems—such as the one shown in Figure 1—typically measure meteorologic conditions such as wind velocity and direction, temperature, light intensity, air pressure, and relative humidity, parameters related to snow drift such as snow particle size and speed at 50 cm above ground level, seismic activity such as ground vibration, and the Earth’s magnetic field. For such setups, autonomy of the remote wireless sensor system must be maximized by minimizing the energy spent on wireless communication. Besides this constraint, the channel characterization was performed without any initial link requirements for data rate in mind. LoRa technology was experimentally tested to determine its operational range, assuming that the data rate could be very low. There is no acknowledgement required or used for the transmissions under study. Transmissions with increasing packet sequence numbers were employed to verify the effective reception of the packets, including validation of the signal-to-noise ratio (SNR) in the chip’s SNR register (if no packet is received, the SNR register is not updated). The directive Yagi-Uda base station antenna only serves to receive sensor data which were transmitted unidirectionally, without acknowledgement, feedback, or adaptive communication to limit energy consumption as much as possible.

Other wireless sensor network technologies could realize the link, but they have some disadvantages compared to LoRa. The following list includes potentially suitable technologies for sub-GHz industrial, scientific and medical (ISM) bands:

- Sigfox
- LTE-M

Sigfox was not chosen due to extreme limitations in payload length, very low data rate, and severe restrictions on the packet rate. Moreover, the ultra-low bandwidth makes the system very sensitive to Doppler shifts. An LTE-M network is currently being rolled out in the US by Verizon. This is a totally new technology, which is still in its infancy.

At both ends of the link, the hardware used for this measurement is the Microchip Technology DM164138 development board [12]. The board relies on a Microchip RN2483 chip. It is capable of transmitting and receiving LoRa signals at 434 and 868 MHz with an output power of 14 dBm and a specified input sensitivity of −148 dBm.

During our measurements, the mobile transceiver is transmitting a packet every five seconds, alternating between both bands. The packets contain sequence numbers, which are registered by the base station transceiver, recording the sequence numbers with the signal-to-noise ratio (SNR) as determined by the RN2483 chip.

The data collected at the transmit side (mobile user) are:

- Time stamp
- GPS coordinates (accuracy ±15 m (50 ft), during 95% of the time)
- GPS altitude (accuracy ±23 m (75 ft), during 95% of the time)

The data collected at the receiver side (base station) are:

- Time stamp
- Sequence number read from the packet
- Band in which this packet was received (434 MHz or 868 MHz)
- Measured SNR for the received packet

### 2.1. Mobile Transceiver

**Figure 2:**
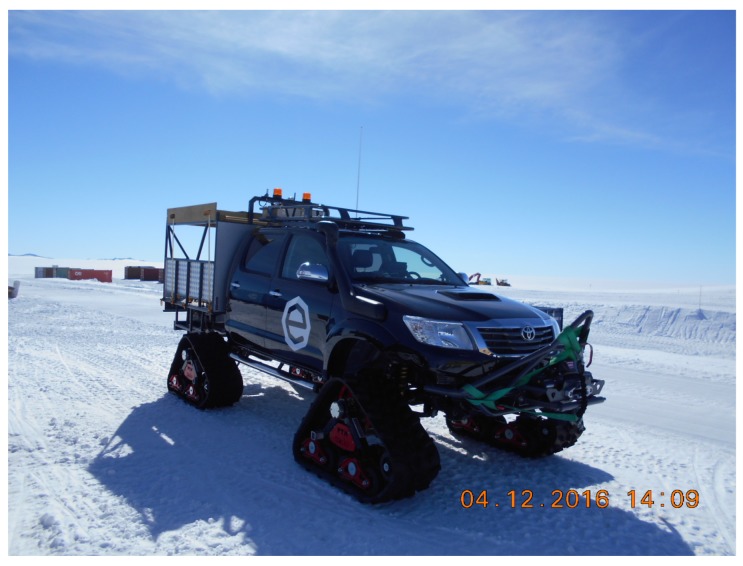
Mobile setup with omnidirectional antennas for the 434 and 868 MHz bands on the 4×4 vehicle.

**Figure 3:**
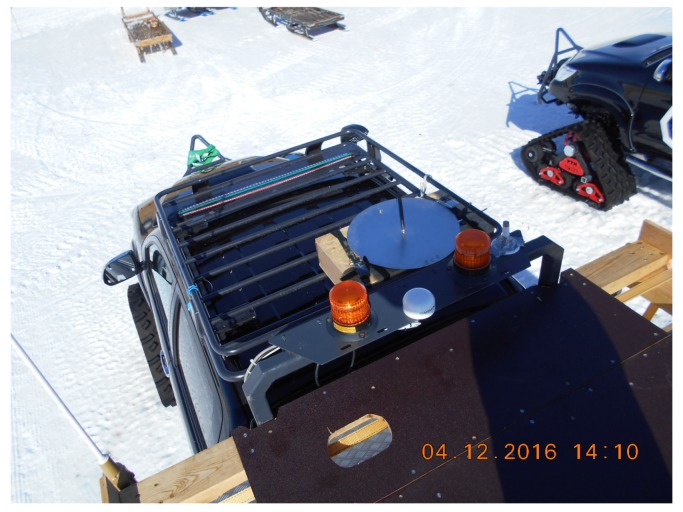
Detail of the 868 MHz antenna mounted on the 4×4 vehicle.

At the mobile side, the LoRa transceiver is mounted in a case, which is placed on top of a snowmobile. The transceiver is connected to an omnidirectional sleeve-dipole antenna for the 868 MHz band, positioned on top of the case. For the 434 MHz band, a similar antenna is mounted on the structure of the cargo frame (see Figure 2 and Figure 3). The mobile LoRa unit is controlled by a Raspberry Pi board [13] via a USB interface. Correct operation of the Microchip Lora module is guaranteed down to $-40{\;}^{\circ }$C, but the Raspberry Pi module contains components that are not certified at this low temperature, making the device unsuitable for permanent Antarctic science experiments. During this campaign, the Raspberry Pi was installed inside the vehicle, where the temperature stays within operational range during the whole expedition. The power for this setup was delivered by a USB battery pack, charged by the vehicle’s power system.

A Python control script running on the Raspberry Pi transmits packets every five seconds, on alternating frequency bands. Each packet contains a unique serial number. Synchronously, a GPS unit in the vehicle records the location and a time stamp. The LoRa transmission parameters are summarized in Table 1. Note that the default coding rate 4/5 was preferred over the more robust 4/8 rate, since the former provides a better trade-off between transmit power required by the energy-constrained nodes and communication reliability.

In general, the tabulated values are the default configuration parameters of the development board, resulting in a data rate of 292.97 bps, transmitted at the maximum available power. The spreading factor produces a link budget of 151 dB and a receiver sensitivity of −137 dBm in ideal conditions [14]. In addition, a cyclic redundancy checksum (CRC) guarantees error-free packets, as only those packets received with a correct CRC will be recorded by the transceiver chip and result in an SNR measurement.

### 2.2. Base Station Transceiver

**Figure 4:**
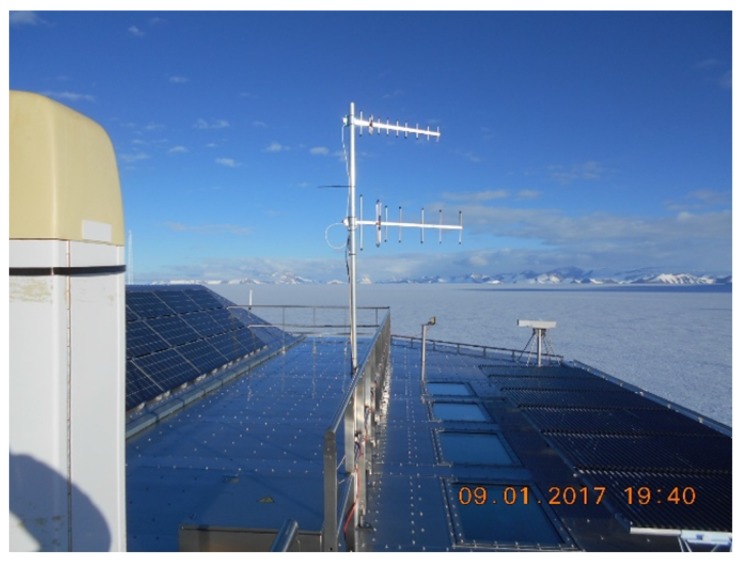
Fixed base station setup with directional antennas for the 434 and 868 MHz bands.

At the Princess Elisabeth Station, two receivers are mounted on the roof, each connected to a Yagi-Uda antenna. The base station height is 1382 m above sea level, with the Yagi-Uda antenna mounted 8 m higher. The type 434 A 434-MHz-antenna, manufactured by LPRS (Low Power Radio Solutions [15]), consists of six parasitic radiators, being one reflector and five directors, besides the folded dipole that acts as a feed. The type 868-914 A 868-MHz-antenna by the same manufacturer contains eight parasitic elements, being one reflector and seven directors, besides the folded feed dipole. Both directional antennas point along the path followed by the mobile transmitter. The trajectory along which the expedition takes place aims to stay within the main beam widths of both directional antennas during the full duration of each experiment. This is possible because expeditions in the Antarctic are performed more or less in a straight line from the base station to the destination, only deviating to avoid cracks in the ice. The base station antennas are shown in Figure 4. The base station receivers are connected to a laptop to record all valid LoRa packets, including the SNRs detected by the receiver, the packet’s serial number, and time stamp.

**Figure 5:**
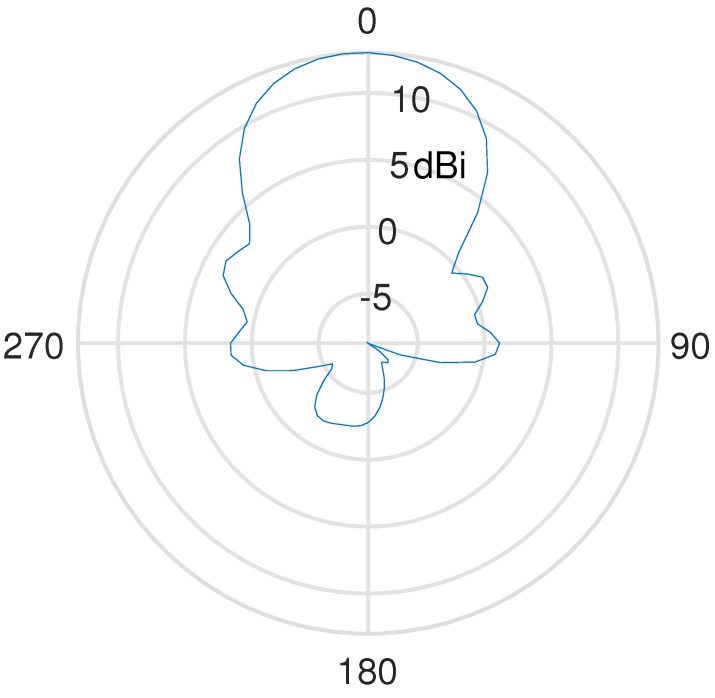
Measured H-plane radiation pattern of the 868 MHz Yagi-Uda antenna.

The radiation pattern of the 868 MHz Yagi-Uda antenna is displayed in Figure 5, showing a −3 dB beam width of $\pm 27.6^{\circ }$ in the H-plane. As for the E-plane, the manufacturer specifies a −3 dB beam width of $42^{\circ }$. At the start of each expedition, the base station Yagi-Uda antennas are pointed in the direction of the path to be traveled, and expeditions aim to always stay well within the main beam’s width. Note that the 868 MHz pattern shown in Figure 5 corresponds to the most narrow beam width. The 434 MHz antenna contains less elements, and therefore has a substantially larger −3 dB beam width, being $50^{\circ }$ in the H-plane and $75^{\circ }$ in the E-plane, as specified by the manufacturer. Hence, alignment errors will have a larger impact in case of the 868 MHz Yagi-Uda antenna. Therefore, it will be more difficult to obtain the maximum antenna receive gain along the path traveled by the mobile user at 868 MHz.

### 2.3. LoRa Modulation

**Figure 6:**
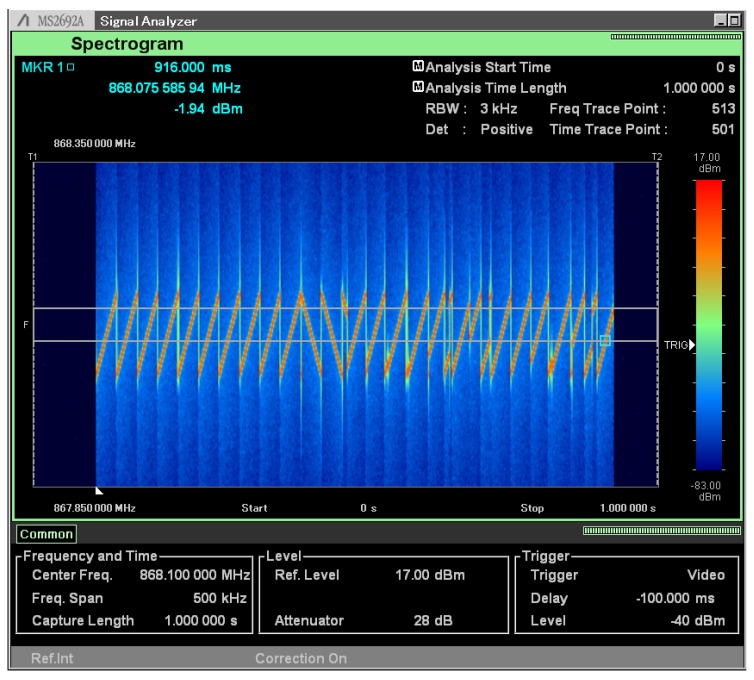
Spectrogram of a LoRa packet. Note the 10 up sweeps, the 2.25 down sweeps of the preamble, and the discontinuities that represent the symbols.

LoRa is based on a patented modulation technology [16] relying on chirp spread spectrum (CSS) modulation. As illustrated by the screen shot of the Anritsu MS2692A spectrum analyzer in Figure 6, the packet starts with 10 up sweeps, followed by 2.25 down sweeps. These down sweeps are the only down sweeps occurring in the transmission. The actual data transmission starts after the 12.25-sweeps-long preamble. Symbols are encoded into constant-rate frequency sweeps, with the information conveyed by a frequency jump. As this trace is generated with a spreading factor of 12, this means that every frequency jump seen in Figure 6 represents 12 bits of data.

To improve the reliability of the transmission, the data are interleaved, spectrally whitened, and gray indexed. Moreover, forward error correction (FEC) is employed.

### 2.4. Calibration

The SN2881 chip employed in the LoRa receiver includes a register for reading out the SNR of the last successfully received packet, returning a value between −128 and 127 dB, according to the documentation. However, measurements have shown that only a small detection range is available. Stronger signals quickly saturate the detector, whereas packets are no longer detected below a given SNR, and hence an SNR value is not available.

**Figure 7:**
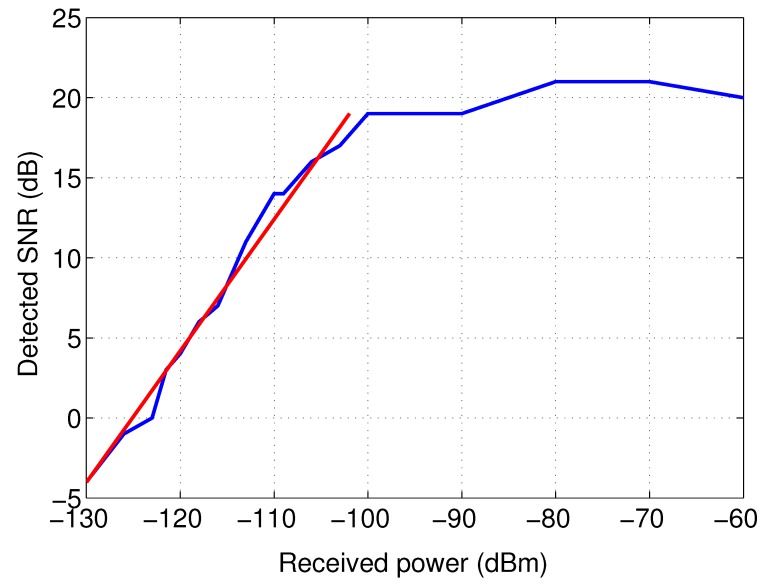
Calibration of the receiver’s signal-to-noise ratio (SNR) measurement.

Additionally, a calibration is necessary to obtain accurate received powers, expressed in dBm. This calibration is performed by connecting the LoRa transmitter, which transmits packets at a power of +14 dBm, via switched attenuators to the receiver. For attenuations in the 100 to 130 dB range, a fairly linear response is obtained in the 868 MHz band. In the case of an attenuation less than 100 dB, the detector saturates. This means that for strong received signals the detector is saturated, resulting in an incorrect SNR measurement, while the packet is received successfully. The calibration results are presented in Figure 7. The calibration procedure yields the following empirical formula to calculate the received power in the −130 to −100 dBm range:

(1) $$\begin{array}{c} P_{dBm}=\frac{{SNR}_{dB}-88.759}{0.8215} \end{array}$$

As the 434 MHz band is received by the same chip and the SNR is measured by the same detector, a similar response as for the 868 MHz band is assumed.

### 2.5. Environmental Factors

We now discuss the potential effect of environmental factors on our experiments. The full weather report during the measurements is listed below:

Weather Information from Princess Elisabeth station
  
Date             : 13/01/2017
Time             : 10,00 UTC
  
Wind Direction   : 84 degrees
Wind Speed       : 11.2 kt
Wind Speed(Max)  : - kt
  
Visibility       : 60 km
  
1st Cloud Layer
Clouds amount    : Ci Sct
         type    : -
       Ceiling   : - ft
2d Cloud Layer
Clouds amount    : -
         type    : -
       Ceiling   : - ft
Humidity         : 52.4 %
Temperature      : -6.2 C
Pressure (QFF)   : 1004.6 hPa
Horizon          : VERY GOOD
Contrast         : VERY GOOD
Present Weather  : NSW
Remarks          : -
  
  
Status of Skiway/Runway: VERY GOOD
  -Last grooming: 10-01-2016
  -Surface condition: Compacted snow on firm base (L 1200M)
  -Visual inspection: 10-01-2016 09:00 UTC
Princess Elisabeth Antarctica
Utsteinen - S71 57’ E23 21’
Antarctica
www.antarcticstation.org

We have observed that environmental factors did not impact the communication in our measurements. Extreme temperatures do not have an effect on the radiowave propagation from antenna to antenna. Receiver performance may be affected by extremely low temperatures, but these were not present at the time of the measurement. The receiving PC and LoRa receiver were mounted in an unheated metal closet, at a temperature slightly above the outdoor temperature, which was $-6{\;}^{\circ }$C. However, the receiver is specified to operate correctly at temperatures down to $-40{\;}^{\circ }$C. Care should be taken that the PC and the microcontroller—which provide the data to the transmitter—are also specified for operation at these low temperatures. The PC that controls the LoRa transmitter was mounted inside the vehicle, where the temperature is $20{\;}^{\circ }$C. The LoRa transmitter itself was mounted on top of the vehicle, at a temperature of $-6{\;}^{\circ }$C. According to the specifications, the transmitter also operates correctly down to $-40{\;}^{\circ }$C.

## 3. Measurement Results

### 3.1. Trajectories

**Figure 8:**
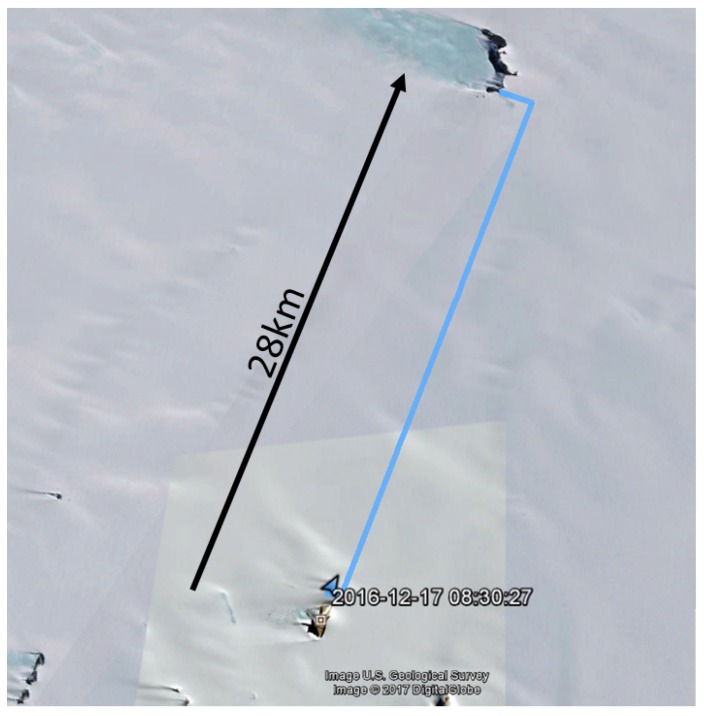
Route from the station Princess Elisabeth Antarctica (PEA) to Vesthaughen followed during the measurements.

In total, data were collected during five expeditions. Two of them applied the dual-frequency setup. At the transmitter side, the GPS locations and their timestamps were recorded, whereas at the receiving side, timestamps and SNRs were recorded for both the 434.1 and 868.1 MHz bands. During both expeditions in which dual-band measurements were performed, the snowmobile drove along nearly straight lines from the Princess Elisabeth station ($71.949960^{\circ }$ S $23.347503^{\circ }$ E) to the first mountain in the northern direction, named Vesthaugen Nunatak ($72.700392^{\circ }$ S $23.554936^{\circ }$ E), at a distance of approximately 30 km, as shown in Figure 8. The expeditions include both a Line-of-Sight (LoS) and a Non-Line-of-Sight (NLoS) path due to the slope of the terrain. No other mountains that could generate reflections are nearby. The main effects are probably caused by changing terrain elevation, often blocking the direct signal path.

In order to know the exact distance between the LoRa transmitter and the LoRa receiver, a GPS unit was installed next to the Lora transmitter on the vehicle. To convert its coordinates to the distance between the antennas, the open-source library Gdal [17] (Geospatial Data Abstraction Library) is used, which translates the WGS 84 angular coordinates, delivered by the Garmin GPS in a GPX format, into distances. For Antarctic regions, we use the projection UTM 34S, containing a valid model for the Earth’s shape in this area.

### 3.2. Received Signals

#### 3.2.1. Line-of-Sight Propagation

**Figure 9:**
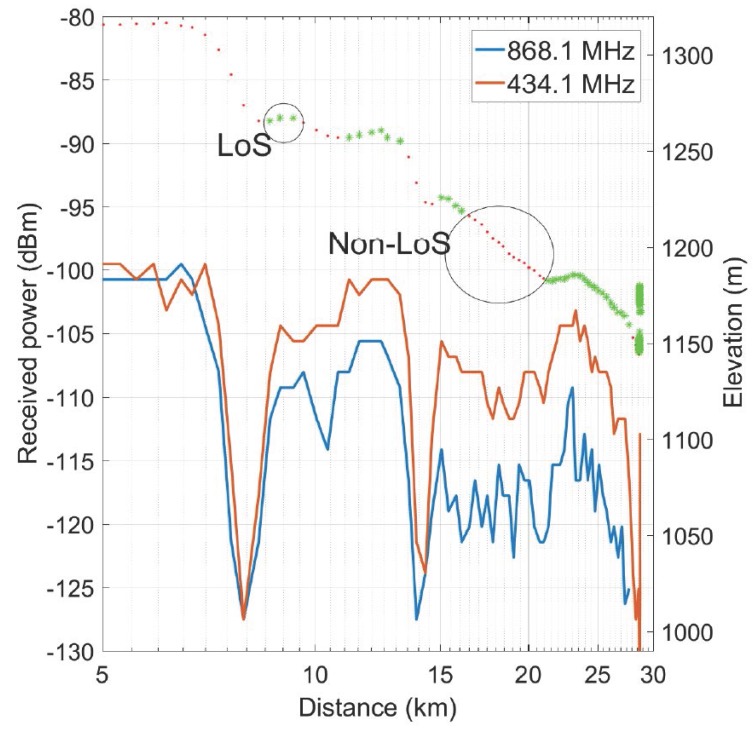
SNR as a function of distance. LoS: Line-of-Sight; NLoS: Non-Line-of-Sight.

The LoS or NLoS conditions are represented in Figure 9 by the top trace, plotting the altitude above sea level, where asterisks indicate LoS operation and dots NLoS conditions with respect to the base station. The bottom part of the graph displays the received power of the transmission as a function of distance. The profile of the terrain is the dominating factor influencing the received signal level. Lower signal powers correspond to locations with no direct LoS, occurring due to a quickly decreasing altitude with respect to the base station. LoS is lost at approximately 7.5 km, 10 km, 14 km, and between 16.5 km and 21 km. Clearly, the smaller decrease in height around 10 km results in a much smaller drop in power compared to the drop around 7.5 km and 14 km produced by a steeper declination. Signals are still received via diffraction around the curvature of the terrain—a phenomenon that is well known to occur in the very high frequency (VHF) and ultra high frequency (UHF) bands [18]. After the dip in elevation, around 21 km from the base station, the altitude of the mobile vehicle increases again, restoring LoS conditions at a distance of 22 km. Note the total absence of other obstacles between the end points of the link, which leaves terrain elevation the only factor directly influencing the propagation.

Due to the varying terrain elevation, the path loss exponent is very difficult to determine. The measured signal drop is about 2 or 3 dB when going from a distance of 20 to 27 km, suggesting a path loss exponent in the range of 1.5 to 2.3 for the log distance path loss model. For comparison, Ref. [19] reports that indoor environments exhibit a path loss exponent of 1.6 to 1.8 in LoS. In free space, the path loss exponent equals 2. For urban areas, the exponent is 2.7 or higher. The snow surface is not actually flat. Therefore, the ground reflection is probably diffuse instead of specular. The path loss extracted from the measurement also indicates this. More measurements are necessary to confirm this assumption.

**Figure 10:**
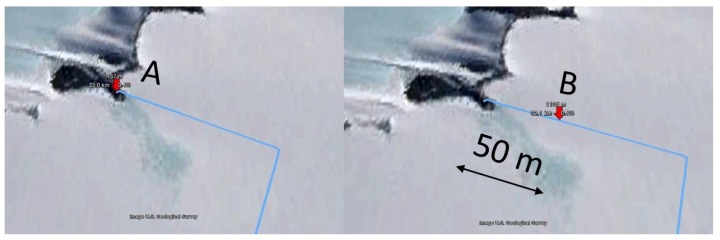
Locations A and B, where the vehicle stopped for a longer period of time.

**Figure 11:**
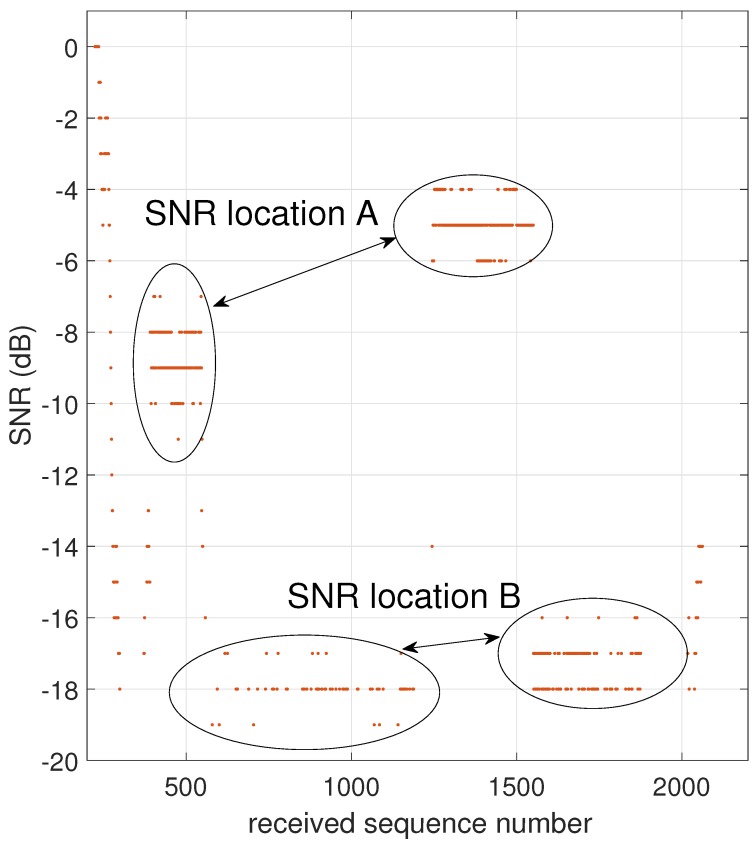
SNR measured at two positions separated by 500 m.

**Figure 12:**
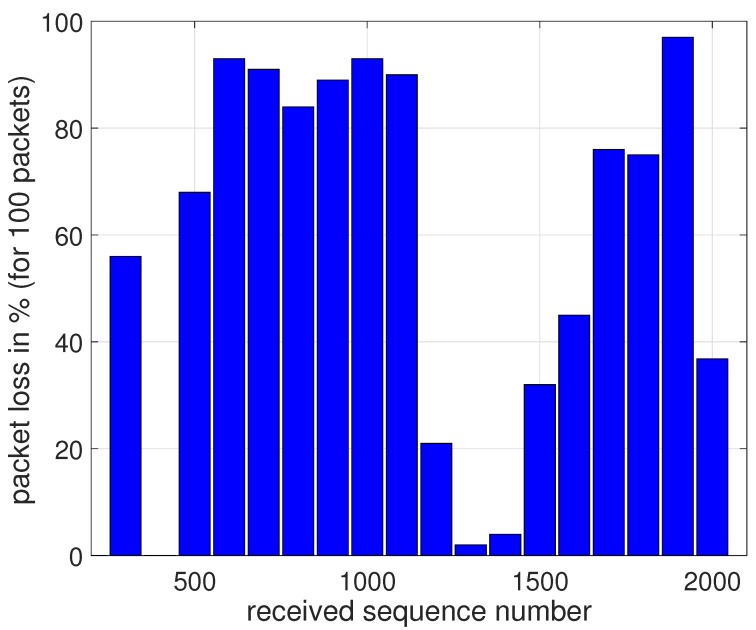
Packet loss observed at two positions separated by 500 m.

At a distance of 28.7 km, large SNR fluctuations are present in the measurement. These fluctuations are caused by the vehicle driving back and forth between two science setups at nearly equal distances from the base station receiver, as shown in Figure 10. Point A is at 1144 m elevation whereas point B is at 1179 m elevation, hence the distance to the receiver changes by only 50 m. In Figure 11, we observe large differences in recorded SNR, but when the vehicle returns to approximately the same spot (A or B), the values are quite similar again. Figure 12 demonstrates that the corresponding packet loss (expressed as the number of packets lost per 100 packets) remains in the same order of magnitude when returning to the same location. These observations confirm the dominating influence of terrain elevation on the radiowave propagation.

The packet loss observed during the LoS measurements is displayed in Table 2. To obtain enough packets for an accurate packet loss calculation, we consider the distance range described in the first two columns. The number of received packets is indicated, together with the percentage of packet loss. Up to a distance of 25 km, not a single packet was lost. Only in the furthest part of the measurement range—between 20 and 30 km—was a limited packet loss of 16.42% recorded. This means that communication is perfect within a 25 km range for a mobile user, and also highly reliable up to 30 km for a fixed user in locations providing clear LoS, such as location A in Figure 11.

A comparison between the 433 and 868 MHz bands reveals a systematically weaker signal for the 868 MHz band. A fairly constant difference between the two frequency bands is observed, with the 868 MHz signal being about 10 dB weaker than the 433 MHz signal. This effect is caused by the differences in path loss, cable losses, and antenna gain. The Friis formula reveals a 6 dB higher path loss for the 868 MHz signal, compared to 433 MHz at the same propagation distance. Yet, the Yagi-Uda antenna 433 MHz exhibits a maximum receive gain of 9 dBi, compared to 13 dBi maximum receive gain at 868 MHz. The additional attenuation experienced at 868 MHz may be explained by the higher cable losses. Moreover, the alignment of the directional antenna plays a role: although the 868 MHz Yagi-Uda antenna provides more gain, it exhibits a narrower beam width, making it more difficult to keep the antenna aligned with the direction of the mobile user. This also explains why the difference in path loss between the two frequency bands is larger than expected.

#### 3.2.2. Non-Line-of-Sight Communication

**Figure 13:**
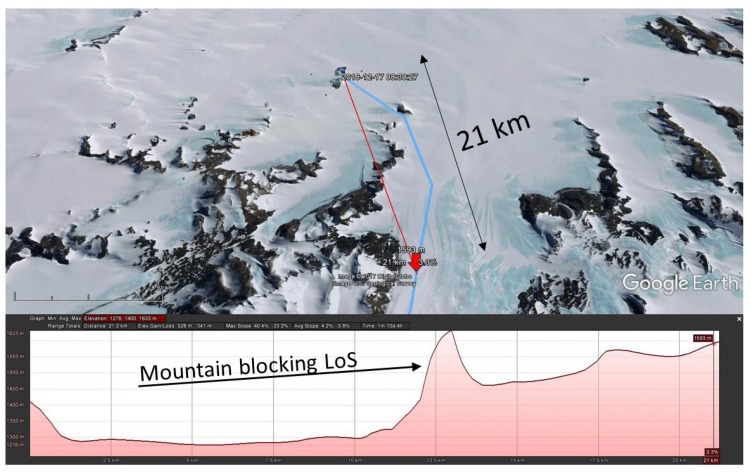
Scenario where NLoS propagation was detected. The location where the transmission took place is indicated on the map.

Two expeditions leaving the Princess Elisabeth station traveled in a southern direction. In this direction, mountains quickly interrupted LoS propagation. In the measurement performed at 868.1 MHz, packets were received up to 21.1 km distance. On the height profile displayed in Figure 13, a sharp mountain is clearly blocking the LoS path. Calculations based on [18] indicate that diffraction is possible. The diffraction loss caused by the mountain top equals 6.88 dB.

On the return trip, exactly the same route was followed. Packets were again received, which demonstrates that the shape of the terrain is the key factor in this transmission. Tropospheric propagation can be excluded, as this mechanism would result in the reception of a more time-varying signal observed over a much larger area.

**Figure 14:**
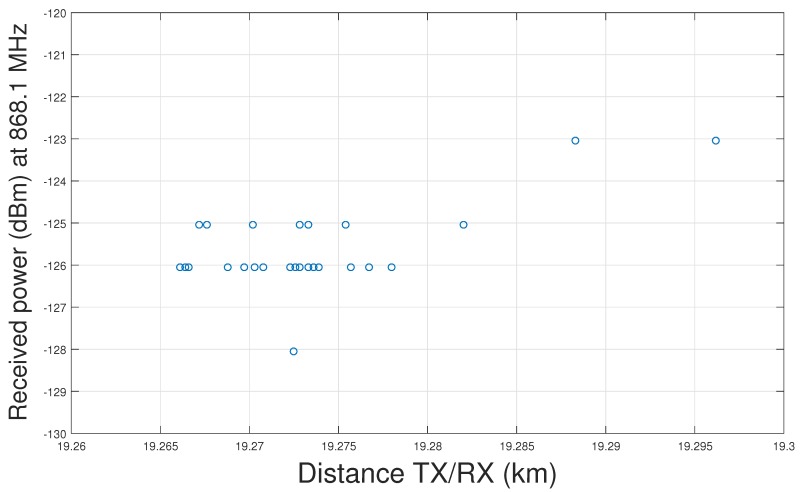
Packets received in NLoS conditions, probably via reflection.

**Figure 15:**
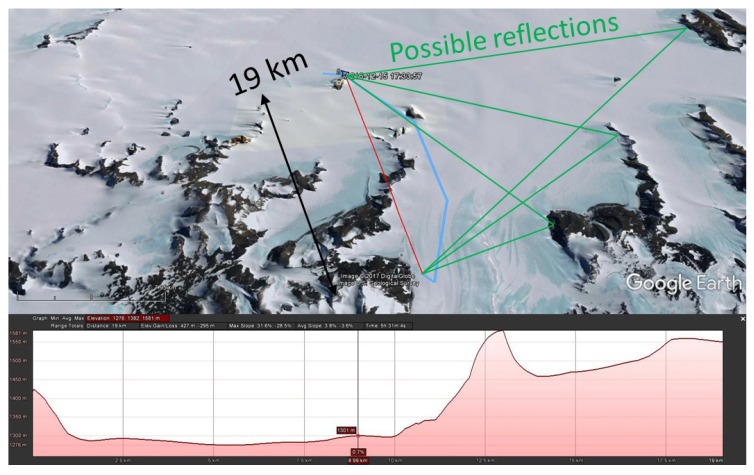
NLoS LoRa communication, with an indication of the potential propagation paths via reflection.

During a different expedition, at a distance between 19.25 and 19.5 km, after a long silent period, suddenly about 200 packets were received, as shown in Figure 14. Figure 15 reveals that there is no LoS path between transmitter and receiver at that location.

**Figure 16:**
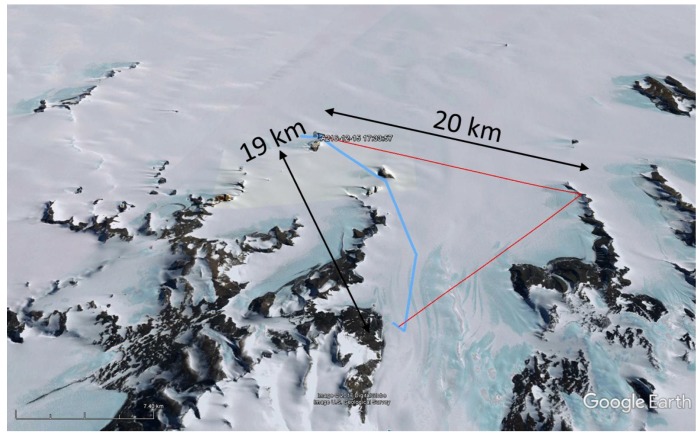
Possible reflection point.

The most likely scenario here is reflection. Finding the exact signal path is difficult, since there are multiple possible routes, as shown in Figure 15. These signal paths are composed of two specular paths, of which one (shown in Figure 16) matches the direction of the base station Yagi-Uda antenna. These observations indicate the possibility of setting up LoRa links without LoS via knife-edge diffraction over mountain ridges, or via reflections on higher mountains that dominate the landscape.

## 4. Discussion

### 4.1. Communication within a 20 km Range

The measurement campaign proves that LoRa modulation is an excellent candidate for long-range low-rate data communication in the Antarctic, within a radius of at least 20 km. During the measurements, directional high-gain antennas were employed at the base station to extend the range. However, the SNR measurement was saturated and no packet loss was recorded at distances smaller than 20 km. Hence, omnidirectional antennas such as a vertical monopole or dipole would still have been sufficient to maintain a reliable link up to 20 km. Larger distances are clearly possible with antennas that provide higher gain. If antenna gain needs to be combined with an omnidirectional radiation pattern, vertical collinear antennas can be used. For example, the X-700HN antenna, manufactured by the Diamond Antenna Corporation [20], provides 13 dBi omnidirectional gain at 434 MHz by means of a collinear array of 11 elements of 5/8λ (the Yagi-Uda antenna used during this measurement campaign provides a gain of 9 dBi).

Note that European or US spectrum regulations are not violated when deploying a high-gain antenna at the receive side only. In all the experiments described in this paper, the mobile transmitter has never used a high-gain antenna. However, in the Antarctic it would be allowed to deploy high-gain antennas at both ends of the link, since there are no specific spectrum regulations that apply to this area.

### 4.2. LoS Communication over Larger Distances

Communication over larger distances is certainly possible in LoS conditions. During the measurement campaign, signals were successfully received at distances up to almost 30 km from the base station. However, due to varying terrain elevation along the trajectory, optical NLoS conditions sometimes occur, resulting in weaker signals and causing some packet loss. Radio waves tend to follow the curvature of the terrain, increasing the range slightly beyond the optical horizon, resulting in continued successful communication, albeit with some packet loss.

At 30 km, the connection was sometimes lost, depending on the position. Driving back and forth between two locations at a 30 km distance, the connection was often restored, indicating the continued influence of terrain elevation. Significantly larger communication ranges should be possible with higher antenna elevations.

### 4.3. NLoS Communication

NLoS communication was observed in a number of measurements at distances of 20 km and more from the base station. The most likely propagation mechanisms are knife-edge diffraction over mountain ridges and reflections on higher mountain tops. Tropospheric effects are not likely, as these occur mainly in lower and mid-latitude regions on Earth. Moreover, the repetitiveness of the phenomenon indicates that propagation is determined by constant factors.

While NLoS propagation is shown to be possible in the Antarctic, the practical setup of such links would require field measurements or ray-tracing simulations to determine suitable antenna locations.

### 4.4. Reproducibility of Measurements

It is very interesting to compare two measurements along the same trajectory performed in different frequency bands. The behavior of the recorded SNR as a function of distance is almost equal at 434 and 868 MHz. This confirms that distance and terrain elevation are the main parameters influencing the propagation. Tropospheric propagation is not expected to have a significant impact, since this propagation mode is occurring mainly at low and middle-latitudes. Therefore, this phenomenon is very unlikely to occur in the Antarctic. In addition, the trajectories followed by the snowmobile are of course only approximately identical. As there are no roads, the vehicle is driving purely on GPS. A slight difference in trajectory did not cause a measurable difference in propagation characteristics, indicating an insignificant influence of multipath propagation.

## 5. Conclusions

In order to directly deploy low-power digital communication with LoRa modulation for the wireless transmission of sensor data to a base station, Line-of-Sight propagation is an essential requirement. If this condition is fulfilled, distances up to 30 km are easily covered with the standard +14 dBm transmit power and an omnidirectional mobile antenna combined with a directive base-station antenna providing a gain of 9 to 13 dBi. The available link budget suggests that communication is also possible by employing omnidirectional antennas at both sides of the link.

The measurement campaign indicates that terrain elevation is the dominating factor influencing the radio propagation, as a Line-of-Sight path can be temporarily blocked due to the curvature of the terrain. Tropospheric radio propagation was not observed, as equal positions resulted in reproducible signal levels over longer periods of time.

Non-Line-of-Sight links are possible, but they require field measurements or ray-tracing simulations to determine the optimal antenna locations. Future research could also focus on the use of other coding gains or on adaptive communication to improve link reliability. However, this will come at the cost of higher power consumption at the energy-constrained sensor node.

## Figures and Tables

**Table 1:** LoRa parameters selected for the measurements. CRC: cyclic redundancy checksum.

Modulation	LoRa
Power	+14 dBm
Bandwidth	125 kHz
Coding Rate	4/5
CRC	On
Preamble length	10
Spreading factor	12
Payload	3 Byte
Frequency 1	868.1 MHz
Frequency 2	434.1 MHz

**Table 2:** Packet loss under LoS conditions.

Start Distance (km)	End Distance (km)	Packets Received	Packet Loss %
0.5	5	219	0.00
5	10	150	0.00
10	15	62	0.00
15	20	95	0.00
20	25	101	0.00
25	30	798	16.42

## Abbreviations

The following abbreviations are used in this manuscript:

CRC	Cyclic Redundancy Check
CSS	Chirp Spread Spectrum
FEC	Forward Error Correction
Gdal	Geospatial Data Abstraction Library
GPS	Global Positioning System
GPX	GPS Exchange Format
IoT	Internet of Things
LoRa	Long Range
LoS	Line-of-Sight
LPRS	Low Power Radio Solutions
NLoS	Non-Line-of-Sight
SNR	Signal-to-Noise Ratio
UHF	Ultra High Frequency
USB	Universal Serial Bus
UTM	Universal Transverse Mercator (projection)
VHF	Very High Frequency
